# Front-End Design for SiPM-Based Monolithic Neutron Double Scatter Imagers

**DOI:** 10.3390/s22093553

**Published:** 2022-05-07

**Authors:** Joshua W. Cates, John Steele, Jon Balajthy, Victor Negut, Paul Hausladen, Klaus Ziock, Erik Brubaker

**Affiliations:** 1 Lawrence Berkeley National Laboratory, Berkeley, CA 94720, USA; vnegut@lbl.gov; 2 Sandia National Laboratories, Livermore, CA 94550, USA; jtsteel@sandia.gov (J.S.); jabalaj@sandia.gov (J.B.); ebrubak@sandia.gov (E.B.); 3 Oak Ridge National Laboratory, Oak Ridge, TN 37831, USA; hausladenpa@ornl.gov (P.H.); ziockk@ornl.gov (K.Z.)

**Keywords:** neutron imaging, neutron double scatter imaging, monolithic scintillation detector, silicon photomultipliers

## Abstract

Neutron double scatter imaging exploits the kinematics of neutron elastic scattering to enable emission imaging of neutron sources. Due to the relatively low coincidence detection efficiency of fast neutrons in organic scintillator arrays, imaging efficiency for double scatter cameras can also be low. One method to realize significant gains in neutron coincidence detection efficiency is to develop neutron double scatter detectors which employ monolithic blocks of organic scintillator, instrumented with photosensor arrays on multiple faces to enable 3D position and multi-interaction time pickoff. Silicon photomultipliers (SiPMs) have several advantageous characteristics for this approach, including high photon detection efficiency (PDE), good single photon time resolution (SPTR), high gain that translates to single photon counting capabilities, and ability to be tiled into large arrays with high packing fraction and photosensitive area fill factor. However, they also have a tradeoff in high uncorrelated and correlated noise rates (dark counts from thermionic emissions and optical photon crosstalk generated during avalanche) which may complicate event positioning algorithms. We have evaluated the noise characteristics and SPTR of Hamamatsu S13360-6075 SiPMs with low noise, fast electronic readout for integration into a monolithic neutron scatter camera prototype. The sensors and electronic readout were implemented in a small-scale prototype detector in order to estimate expected noise performance for a monolithic neutron scatter camera and perform proof-of-concept measurements for scintillation photon counting and three-dimensional event positioning.

## 1. Introduction

**Figure 1:**
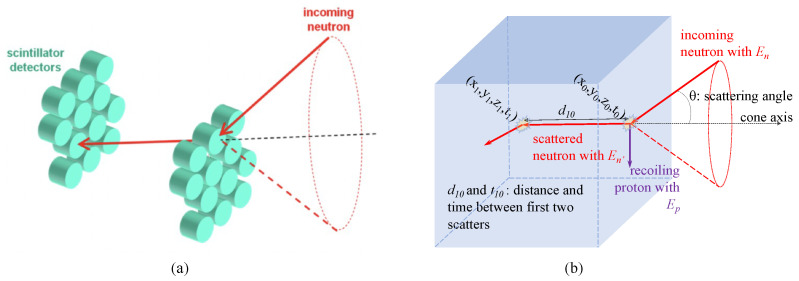
Neutron double scatter imaging is illustrated in (**a**), for a traditional detector configuration, comprising two planes of cylindrical organic scintillator detectors. The kinematics of neutron elastic scattering allow the incidence angle of neutrons that scatter in both detector planes to be constrained to conic volumes, where successive coincidence measurements facilitate source localization through spatial correlation of overlapping projections [1,2]. A neutron double scatter camera, conceptualized from a monolithic block of plastic scintillator, is illustrated in (**b**). In this detector configuration, coincidence detection efficiency, and thereby imaging sensitivity, can be increased by more than an order of magnitude. Localizing neutron scatter sites through resulting spatiotemporal distributions of generated scintillation light provides the kinematic information for image reconstruction [3].

Emission imaging of radionuclides with fission-energy neutron signatures can be used to locate and characterize neutron-emitting sources of interest, such as special nuclear material. Consequently, this imaging modality has potential value in a range of nuclear non-proliferation applications. Neutron emission imaging can be accomplished with coincidence detection of sequential elastic scatter interactions in organic scintillator arrays, as depicted in Figure 1a. By recording time of interaction and deposited energy for both events in different detectors, neutron energy after the first interaction can be determined, and the initial trajectory of incident neutrons can be confined to a conic shell with dimensions defined by the position, time, and energy resolution of the scintillation detector array. The overlap of many neutron scatter cone projections will correlate with the position of a fast neutron source in the imager’s field of view (FOV) [1,2]. A challenging aspect of neutron double scatter imaging is the low coincidence detection efficiency, resulting from the relatively low interaction probability of fast neutrons in organic detector media in combination with double scatter geometric acceptance of a finite array of cells, as depicted in Figure 1a.

One approach for increasing imaging efficiency for neutron double scatter imagers is to employ a monolithic detection volume for the scatter camera, as depicted in Figure 1b. This detector configuration aims to maximize the detector’s geometric efficiency by exploiting neutron scatter events occurring in a single, large detection volume. Since the mean path length between scatter events for neutrons of a few MeV in energy is on the order of centimeters, a single organic scintillator of moderate size could feasibly be used for the detection of multiple neutron scatter events for fast neutron imaging. To accomplish this, scintillation light can be read out on one or more sides of the detection volume with position sensitive photosensor arrays, and interaction times and positions for sequential neutron scatter events would be derived from the spatiotemporal distribution of scintillation light from both events at the sensor array. Owing to the fact that the average time between neutron interactions is similar to the decay time for the luminescence kinetics of fast organic scintillators (on the order of a few nanoseconds), separate estimations for three dimensional position and time of interaction can be derived for each scatter event. Thus, by exploiting time-resolved neutron and scintillation detection kinematics, this detector configuration can overcome detection efficiency and resolution limits imposed by neutron double scatter cameras comprising arrays of organic scintillation detectors instrumented with a single channel of readout. Moreover, this is accomplished in a compact form factor where detection efficiency and achievable resolution can be optimized in mobile or transportable detector designs.

This single, monolithic detector approach to neutron scatter imaging can require relatively large arrays of pixelated photosensors to be instrumented and coupled to the sides of a monolithic volume of organic scintillator. Silicon photomultiplier (SiPM) arrays are good candidates for this approach, as they offer several key advantages, such as high optical photon detection efficiency (PDE), fast time response and single photon time resolution (SPTR) [4,5], availability in a large variety of pixels sizes for optimizing detector response, and the fact that they are assembled from discrete photosensor elements from which inter-channel optical and electrical crosstalk can be isolated with proper detector design. However, there are also potential performance tradeoffs with integrating SiPM arrays into a scintillator monolith for double scatter imaging. Primarily, the uncorrelated and correlated noise rates can be orders of magnitude higher than for vacuum photosensors [6]. Since the monolithic neutron double scatter concept exploits light maps incident upon photosensor arrays, these noise events could create nuisance photon statistics which could degrade position and timing estimates for neutron interactions and thereby achievable imaging performance and image quality.

In this work, we perform fundamental sensor performance measurements to evaluate the potential for developing SiPM-based, monolithic neutron double scatter cameras. After down-selection to an SiPM of interest, expected uncorrelated and correlated noise rates were quantified as a function of applied overvoltage. Achievable SPTR was also investigated for different data acquisition and digital filtering methods. Finally, a small scale prototype detector was constructed to make an evaluation of expected noise performance and demonstrate 3D positioning capabilities from photon counting with a single detection volume instrumented with multiside readout of SiPM arrays. Altogether, we demonstrate that with proper sensor selection and tuning of readout and data analysis, SiPM arrays provide a viable option for construction of a monolithic neutron double scatter camera.

## 2. Materials and Methods

### 2.1. Silicon Photomultiplier Selection

**Figure 2:**
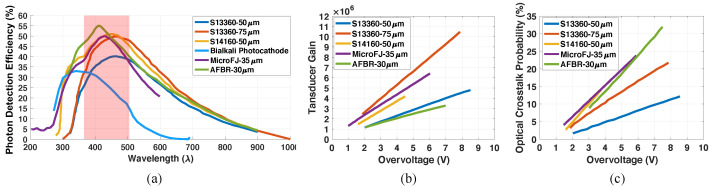
Photon detection efficiency, transducer gain, and optical crosstalk probability for various commercial silicon photomultipliers [7,8,9,10] (manufacturer’s data) are shown in (**a**,**b**,**c**), respectively. A red region is also highlighted in (**a**), denoting the luminescence emissions band of EJ-204 plastic scintillator [12].

A fully realized single volume neutron double scatter camera will leverage the detection of scintillation photons sparsely distributed over SiPM arrays. Thus, the desired function of the sensor array is to record the time of arrival, intensity, and location of light photons from interactions in the scintillator monolith. SiPMs have advantageous characteristics for this approach, including high photon detection efficiency (PDE), high gain, compact footprints with low voltage operation, and capability of fast time response. In Figure 2a, we compare the PDE of Hamamatsu S13360 sensors with 50 and 75 µm cell size, Hamamatsu S14160 SiPMs with 50 µm cell size, OnSemi MicroFJ SiPMs with 50 µm cells, and Broadcom AFBR-S4N SiPMs with 30 µm cells, plotted as a function of wavelength, at the vendor’s suggested operating voltage [7,8,9,10]. The quantum efficiency of a Hamamatsu multi-anode photomultiplier (MAPMT) with a bi-alkali photocathode is shown for comparison [11] (equivalent to PDE, as reported in Figure 2). The emissions region of a fast plastic scintillator, Eljen EJ-204 [12], is also highlighted in red. The SiPMs offer an overall advantage of higher photon detection efficiency in this region of interest.

The sensor array should also be capable of resolving individual light photons, such as to accurately record the time arrival profile of the luminescence yield. Thus, sensors need to have sufficient gain to facilitate single photon counting. Figure 2b shows transducer gain versus applied overvoltage (voltage applied above sensor breakdown voltage $V_{br}$) for the same sensors outlined above, where gain increases with applied bias. High sensor gain can also play a key role in achievable single photon time resolution (SPTR), where a good signal-to-noise ratio is required to minimize the influence of electronic noise. Unfortunately, uncorrelated noise discharge from thermionic emissions (dark counts) and correlated noise photons from these discharges (optical crosstalk) also increase with applied overvoltage, as depicted in Figure 2c. These noise events will contribute to photon statistics employed for deriving 3D event information with photon counting procedures to quantify the spatiotemporal scintillation light distributions from monolithic detector volumes instrumented with multi-side readout. Thus, excessive uncorrelated and correlated noise photons can contaminate estimators for position, energy, and time of interaction and degrade neutron double scatter event information and ultimately achievable image quality. To balance these two basic requirements, sensors that achieve sufficient gain for single photon counting while also minimizing noise photon production are optimal candidates for integration into a monolithic scatter camera. Considering the data presented in Figure 2a,b, we selected the Hamamatsu S13360 SiPM with 75 µm cell size for further evaluation.

### 2.2. Silicon Photomultiplier Noise Characterization

**Figure 3:**
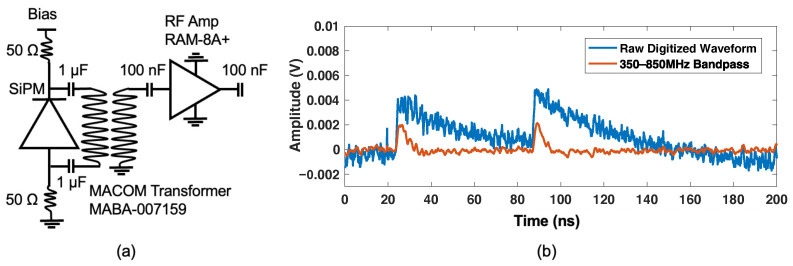
A schematic of the front-end readout circuit used for evaluation of Hamamatsu S13360-6075 SiPMs is shown in (**a**). An example, raw digitized trace from dark counts from a SiPM, along with the same trace with a 350–850 MHz bandpass, is shown in (**b**).

While manufacturer data provide an initial comparison between the sensors outlined in Figure 2, the chain of correlated noise photon generation and detection can be complicated [13]. To quantify the expected noise photons in a single volume neutron double scatter camera employing SiPM arrays for light readout, we performed noise characterization studies on a Hamamatsu S13360-6075 sensor (6 × 6 mm${}^{2}$ active area). In Figure 3, we show the front-end readout circuit used for the noise characterization measurements, consisting of a low noise and high frequency circuit similar to that described in [4,5] but reduced to a single RF amplifier. SiPMs were differentially connected to a balun transformer, which passively boosts signal by a factor of two in a balanced-to-unbalanced configuration. Signals were amplified by a Minicircuits RAM-8A+ radio-frequency (RF) amplifier [14] and passed to a DRS4 evaluation board [15], where they were digitized at 5 Giga-samples per second (GSa/s).

Total correlated and uncorrelated noise rate was quantified by using a “line trigger”, which has no correlation with the noise events of interest. Thus, each trigger represents a random 200 ns sampling of the SiPM output. The noise content in each trace can then be used to evaluate the statistical contribution of these events to acquisitions in a real application. Digitized waveforms were recorded and analyzed in post processing. Waveforms were processed with a 350–850 MHz bandpass, as depicted in Figure 3b, and a peak-finding algorithm was used to count uncorrelated and correlated noise photons. These measurements were performed as a function of overvoltage, thereby quantifying the noise performance with different applied bias.

### 2.3. Single Photon Time Resolution Measurements

**Figure 4:**
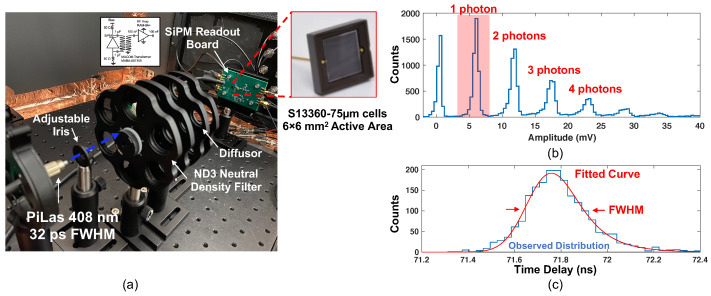
The experimental setup used to measure single photon time resolution with a fast laser is shown in (**a**). Waveforms from the detector readout shown in (**a**) were digitized and analyzed in post processing, where single photons in the detector were filtered, as illustrated in (**b**). An example histogram of time difference between the laser external triggering and the time pickoff on the SiPM waveforms is shown in (**c**), where the distribution is fit with a convolution of Gaussian and exponential curves, as outlined in [17].

The formulation of neutron event reconstruction also requires precision arrival time information for scintillation photons. We evaluated the achievable SPTR for the S13360-6075 SiPM using the experimental setup shown in Figure 4a. An Advanced Laser Diode Systems Picosecond Injection Laser (PiLas) (408 nm peak wavelength) was configured with 30% intensity and 100 kHz repetition rate. With these settings the temporal width of light pulses were measured to be 32 ps FWHM with a streak camera [16].

Detector signals were recorded with two different waveform digitizers. The first was a LeCroy Wavemaster 806ZI-B, which digitized the signals at 40 GSa/s and 3.5 GHz. The second was a DRS4 evaluation board. The digital oscilloscope served as a gold standard comparison, and the DRS4 evaluation board served as a proxy for the DRS4-based SCEMA boards proposed for readout of a prototype neutron double scatter camera [18]. Digitized waveforms were processed offline. Detector signals were fit with a cubic spline, and the time delay between the reference trigger from the laser and the detector was calculated as a function of threshold. An example SiPM pulse height distribution from laser irradiation is shown in Figure 4b. Single photon events were selected as the 6σ width around the “1 photon” peak. These events were processed, and an example resulting distribution is shown in Figure 4c. This distribution was fit with a Gaussian convolved with an exponential function, as outlined in [17].

### 2.4. Small-Scale Prototype Design, Fabrication, and Evaluation

**Figure 5:**
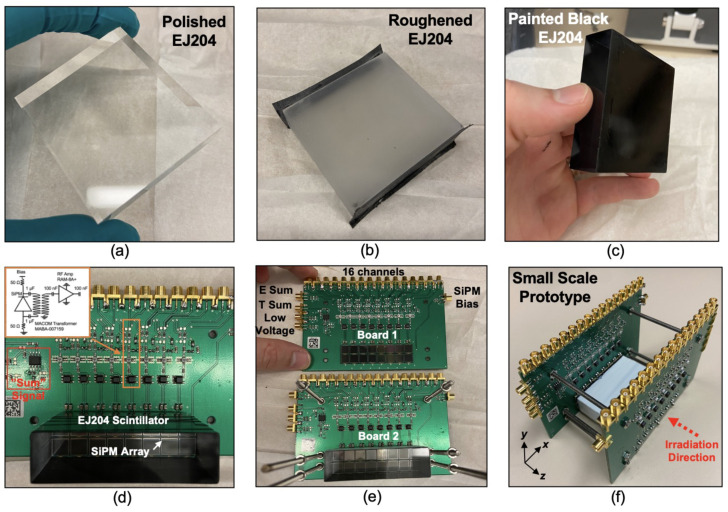
A piece of polished 17 × 55 × 50 mm${}^{3}$ EJ-204 plastic scintillator is shown in (**a**). The four “side walls” of the scintillator were roughened and painted black, as shown in (**b**,**c**). This scintillator block, optically coupled to a prototype readout board is shown in (**d**). Two of these readout boards (**e**) were used to read out the scintillator block from both ends, as depicted in (**f**).

In order to empirically derive the expected optical photon crosstalk in an SiPM-based monolithic scatter camera, a small-scale prototype detector was constructed and evaluated, as depicted in Figure 5. A 55 × 50 × 15 mm${}^{3}$ EJ-204 scintillator was roughened with sand-paper (50 grit) and painted black, as depicted in Figure 5a–c, where two “exit surfaces” remained polished. This scintillator block was coupled on the polished “end surfaces” to SiPM arrays comprising 2 × 8 arrangements of S13360-6075PE SiPMs. Each channel of the SiPM array has the electronic readout circuit outlined in Figure 3a, with the RAM-8A+ RF amplifier employed. The readout board also includes a multiplexed “sum signal” which can be used for triggering, instrumented with a summing amplifier after the RF amplifier. Each channel of the detector shown in Figure 5 was connected to separate channels of a CAEN V1742 [19] multichannel digitizer, which digitized a 200 ns capture of detector waveforms at 5 GSa/s. The “sum signal” from both sides of the small-scale prototype detector were combined to form a global energy channel which was used to trigger waveform capture after leading edge discrimination. The leading edge threshold was adjusted so that it was just above observed baseline noise in the global energy channel.

This small-scale prototype detector and data acquisition were used to quantify the expected total uncorrelated and correlated noise photon population in a fully instrumented 5 × 5 × 5 cm${}^{3}$ detector using the same procedure outlined in Section 2.2 applied to each of the 32 channels of the prototype detector. Measurements were also performed to demonstrate the ability to count optical photons arriving at the SiPM arrays in the small scale prototype, derive energy imparted to the detection volume, and estimate event position of interaction in three-dimensions. For this, an experimental setup was configured such that a 10 µCi Cs-137 source irradiated only half of the prototype detector’s scintillation volume by shielding half of the detector with a 5 cm thick tungsten block.

## 3. Results

### 3.1. Silicon Photomultiplier Noise Characterization

**Figure 6:**
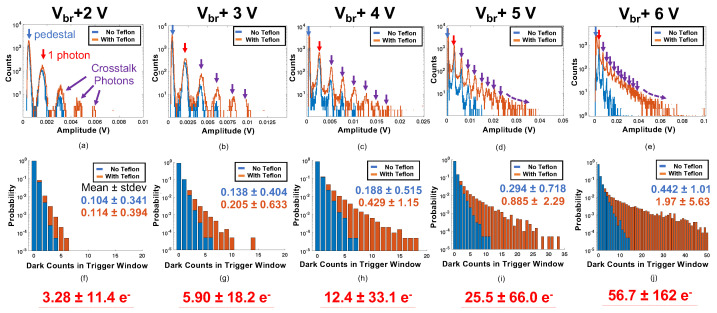
Dark noise spectra for a Hamamatsu S13360-6075CS SiPM as a function of overvoltage (increasing 2 V to 6 V from left to right) is shown in the above figures (**a**–**e**). The same distributions, converted into number of noise counts/photons, is shown in the figures below (**f**–**j**), along with point estimates for mean and standard deviation of the distributions. Below each configuration, the mean number of expected noise photons within a 15 ns region of interest (taken from point estimates in (**f**–**j**)), for six sided readout of the same sensor and operating voltage, is shown in red.

Measured dark counts and optical crosstalk photon spectra are shown in Figure 6a–e as a function of applied overvoltage. Noise photon distributions for measurements where the sensor’s active area was bare are shown in blue, and measurements with the active area wrapped in several layers of Teflon tape are shown in orange. A blue arrow also indicates the pedestal photon peak (when no noise photons were observed in the waveform capture), and a red arrow indicates the single noise photon peak. Using the sensor’s single photon amplitude, the noise photon distributions in Figure 6a–e are represented in “number of photons” in Figure 6f–j. Point estimates for the mean and standard deviation of the number of noise photons in each 200 ns waveform capture are also shown.

### 3.2. Single Photon Time Resolution (SPTR) Measurements

**Figure 7:**
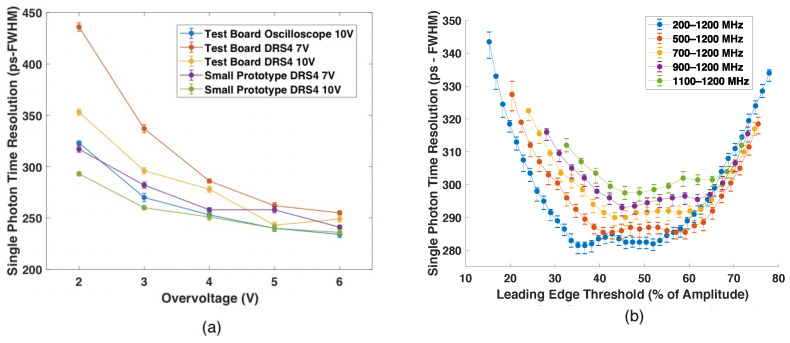
Measured single photon time resolution for Hamamatsu S13360-6075 SiPMs with readout instrumented in both a test board and also in a small scale prototype readout is shown in (**a**). The SPTR, plotted as a function of different digital bandpass filters is also shown in (**b**).

SPTR for both the S133360-6075CS SiPM with readout instrumented on the test board and also for the S13360-6075PE package on the small-scale prototype readout board is shown in Figure 7a, as a function of applied overvoltage. The baseline measurement (blue) with a fast digital oscilloscope achieves 275 ± 5 ps FWHM at the manufacturers recommended overvoltage of 3 V. The orange and yellow curves show the SPTR for the 6075CS package measured with the test board using a DRS4 evaluation board at applied RF amplifier voltages of 7 and 10 V, respectively. The purple and green curves show measured SPTR for the 6075PE package on prototype readout boards shown in Figure 5d–f.

Figure 7b shows the measured SPTR for a single pixel on the small scale prototype board (Figure 5d) operated at $V_{br}$ + 3 V with 7 V applied to the RF amplifier and digital bandpass filters applied having lower frequency cutoffs ranging from 200 to 1100 MHz (constant upper frequency cutoff of 1200 MHz). Increasing the lower frequency cutoff reduces the overall width of the voltage signal and thus improves the ability to resolve multiple optical photons detected at the sensor close in time. However, it also reduces the signal to noise ratio and thereby increases the influence of electronic noise on achievable SPTR.

### 3.3. Small-Scale Prototype Evaluation

**Figure 8:**
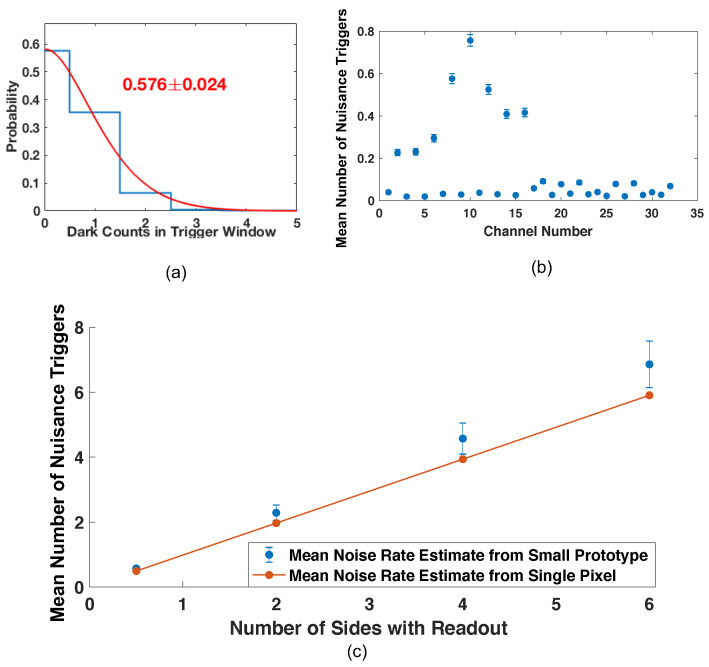
Example distribution of the number of “nuisance” or noise photons recorded for a channel of the small scale prototype detector is shown with a Poisson fit in (**a**). Mean number of nuisance photon triggers is plotted for each channel in (**b**). Estimated number of noise photons in a 15 ns region of time is shown as a function of the number of sides of a 5 × 5 × 5 cm${}^{3}$ monolithic scintillator block instrumented with detector readout us shown in (**c**).

An example histogram of the number of noise photons observed within a 200 ns window, for one of the small prototoype’s 32 channels, is shown in Figure 8a. Shown also is a Poisson fit to the observed distribution, along with the distribution’s mean and standard deviation. These data were taken with an applied overvoltage of 3 V, which was selected from data represented in Figure 6 and Figure 7 to balance photon counting capabilities, noise photon population, and achievable SPTR. The means and standard deviations of the noise photon distributions observed for all 32 channels of the prototype are shown in Figure 8b. Figure 8c plots the mean number of expected noise or “nuisance” photons which would be present in a 15 ns region of interest or photon counting window for data analysis as a function of the number of sides with which a 5 × 5 × 5 cm${}^{3}$ is instrumented with an identical SiPM array fully covering each face. Note that the first data point along the x-axis of Figure 8c is at “0.5 sides with readout”, as the two 16 channel arrays employed in this noise measurement comprise at most half of one side of readout for a 5 × 5 × 5 cm${}^{3}$ detector module.

**Figure 9:**
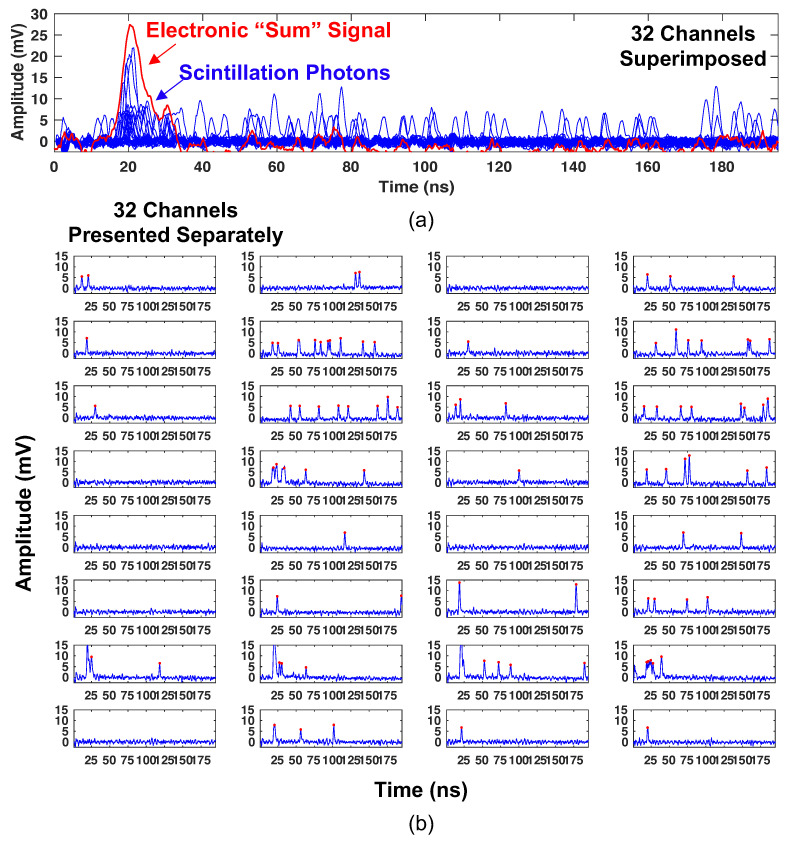
An example of spatiotemporal photon distribution of optical photons arriving at the sensors in the small prototype detector readout is shown in blue in (**a**), along with the “sum trigger” in red. The same data, separated into each electronic channel, is shown in (**b**), along with red dots that indicate the peak location of photons.

Figure 9a shows an example of the photon counting procedure for a single energy deposition, where all 32 channels of the small scale prototype are superimposed. The digitized waveform from the sum trigger, for the same event, is shown in red. Note that the amplitude of the optical photon pulses in Figure 9a exhibit the discrete nature of Geiger cell discharges (with variation in avalanche gain). Thus, the front-end signal processing outlined in Section 2.1 facilitates time-resolved optical photon counting of the scintillation light distribution at the SiPM arrays. The density of optical photons also correlates with the “sum trigger” from all channels (red). However, it is important to note that this “sum” signal is derived from an analog sum of the detector timing signals on the front end of the electronic readout boards, which is subject to resistive attenuation and bandwidth limitation through the summing amplifier. Thus, the sum signal is useful for event triggering of data acquisition, but it does not represent a true digital sum of the fast timing signals (which can be accomplished separately from analyses of digitized waveforms). Figure 9b shows the detected optical photon distributions for all thirty-two channels displayed separately, where red dots represent the output of a peak finding algorithm used for optical photon counting. These data show that instrumenting a monolithic scintillator with arrays of discrete photosensor elements, each with appropriate front-end signal processing, facilitates optical photon counting. This procedure can be exploited to derive time, energy, and position of interaction estimates for ionizing radiation events in the detection volume.

**Figure 10:**
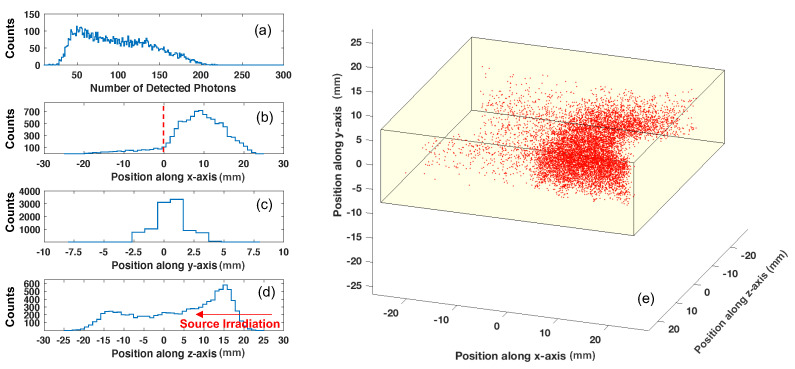
Energy spectrum from Cs-137 irradiation of the small scale prototype, calibrated to the number of detected photons is shown in (**a**). The reconstructed center of gravity positions of interaction along the x, y, and z axes are shown in (**b**,**c**,**d**), respectively. The red vertical dashed line in (**b**) indicates the edge of the tungsten block that was used to shield the x < 0 region of the scintillator. In (**e**), a three-dimensional plot of the calculated positions of interaction is shown.

Figure 10 shows energy and position measurement from the collimated Cs-137 measurements. The resulting energy distribution, recorded as the number of detected photons, is shown in Figure 10a, which exhibits a Compton continuum and a shoulder beginning at approximately 140 detected optical photons, for this small-scale prototype. Figure 10b–d show histograms of the calculated light-intensity weighted position of interaction along the x, y, and z axes, respectively. The calculated positions of interaction are displayed in a three-dimensional scatter plot in Figure 10e, along with surfaces defining a cuboid with dimensions matching that of the plastic scintillator. The calculated positions of interaction exhibit the expected collimated response along the x axis.

## 4. Discussion

SiPMs provide attractive characteristics for implementation in large area, discrete element photosensor arrays for prototype single volume neutron double scatter cameras. With proper signal conditioning, they facilitate optical photon counting, which can be exploited to map spatio-temporal light distributions at sensor arrays for 3D position of interaction estimation, event time pickoff, and energy deposited for each interaction. Unfortunately, they also exhibit high dark count rate and correlated optical photon emission compared to vacuum photosensor technologies, which could potentially influence these estimates for detected events. Moreover, coupling large arrays of SiPMs to optically transparent media allows correlated noise photon emissions that escape active elements of SiPM channels to be detected in adjacent or opposing sensor elements, thereby increasing the total noise photon population in these event estimates. In this work, we have compared relevant characteristics of several commercial SiPMs for this application, down-selected to a candidate sensor with optimal characteristics (Hamamatsu S13360 with 75 µm cell size), and evaluated achievable noise performance and SPTR with this candidate SiPM.

### 4.1. Sensor Noise Analysis

Total noise rate (uncorrelated dark counts and correlated optical crosstalk) for a Hamamatsu S13360-6075 SiPM (6 × 6 mm${}^{2}$ active area) was quantified as a function of applied overvoltage. These measurements were performed with the active area of the sensor bare and also covered in several layers of Teflon tape. There is a well known increase in optical crosstalk photon detection when one or more SiPMs are optically coupled to a scintillation element [20], as crosstalk photons that escape into the detector volume can be reflected at the scintillator boundaries and detected in the same SiPM or other SiPMs in an array. In the context of a single volume neutron scatter camera, where a scintillator monolith would likely be surrounded on multiple sides with SiPM arrays, the detection of optical crosstalk photons from opposing sensor array elements could increase noise photon population and potentially influence estimations on position, energy, and time of interaction. Thus, we performed these single pixel measurements to understand what the expected noise photon rates would be for “best” and “worst” case scenarios (bare sensor versus covered in reflector). Measurements with the SiPM covered in reflector represent a case where optical crosstalk photons have the highest probability of being back reflected and detected and thereby represent the limit in expected noise rate.

For operating voltages of $V_{br}$2 V to $V_{br}$ + 6 V, the mean number of noise photons in a single pixel, observed in a 200 ns photon counting window, ranged from 0.114 to 1.97. As an example, a 5 × 5 × 5 cm${}^{3}$ organic scintillator covered on all six sides by arrays of S13360-6075 SiPMs would be made up of 384 channels. A point estimate of the total expected mean noise rate from these data, in the worst case (measurement with reflector at highest overvoltage of $V_{br}$ + 6 V), would be 56.7 ± 162 optical photons within a 15 ns photon counting window. However, these data may not fully account for more complex optical photon crosstalk detection chains that could arise from large sensor arrays coupled to a monolithic detection media, which is more accurately reflected in the small scale prototype noise estimates (Figure 8). The results of this study, unsurprisingly, indicate that both the operating point of SiPMs and channel density must be balanced against achievable lower level threshold for detection, as this will ultimately influence imaging efficiency. With this in mind, the lowest overvoltage that achieves sufficient signal-to-noise ratio to enable accurate identification and counting of individual optical photons should be used. For the SiPM and circuit studied here, this was accomplished at $V_{br}$ + 2 V, where the lower sensor gain only produced a 9.62% increase in total noise rate between unreflected and reflected measurements. However, sensor gain also influences achievable timing performance in an optical photon counting detector, and there are additional considerations for achievable timing performance, which we detail below.

### 4.2. Single Photon Time Resolution

Time of interaction for each neutron scatter event would conceivably be derived from detection time of the earliest scintillation photons. Therefore, SiPMs for monolithic neutron scatter cameras should also exhibit good SPTR, which is influenced by both the sensor architecture and also electronic noise. The latter is influenced by both the large terminal capacitance of SiPMs and also the performance of front-end readout components. In fact, for large area SiPMs, the influence of electronic noise can dominate achievable SPTR [21] if readout electronics are not appropriately implemented [2,3]. In this work, we used the passive signal gain from a balun transformer in a balanced-to-unbalanced configuration in combination with a low noise and high gain RF amplifier. The SPTR for a S13360-6075 SiPM was measured with this readout using a fast laser and digital oscilloscope (40 GSa/s and 3.5 GHz bandwidth) or various implementations of the DRS4 chip (5 GSa/s and 900 MHz bandwidth). Thus, we studied what was the limit in achievable configuration given the SiPM and front-end readout selection and also what is achievable with data acquisition which can scale to prototype monolithic scatter cameras.

A first set of measurements explored achievable SPTR as a function of overvoltage using different values of applied voltage to the RF amplifier with the different waveform digitizers. Measured SPTR ranged from approximately 250 ps to 450 ps FWHM depending on applied overvoltage and readout configuration, which make this sensor and readout combination competitive against multi-anode photomultiplier alternatives [11] for this application. As expected, the best SPTR was achieved with the digital oscilloscope and highest amplifier voltage supply. A moderate performance tradeoff was observed with the DRS4-based readout and lower operating voltage with a small test board. However, the SPTR measured with a channel of the small-scale prototype detector (Figure 5), where the electrical design was more carefully controlled, exhibited lower electronic noise and only a small performance tradeoff, as compared to the digital oscilloscope. Adjusting the RF amplifier’s supply voltage from 10 V to 7 V reduced total power draw, which exhibited a small performance tradeoff of 8.46% in the prototype readout board with DRS4 acquisition. Based on these measurements, we find this sensor, readout circuit, and digitizer combination yields acceptable SPTR for a prototype monolithic scatter camera. The conclusions of the sensor noise analysis (Section 3.1) suggest the lowest possible overvoltage for which single scintillation light photon pulses can be observed should be used in a prototype detector design. However, as shown in Figure 7, higher overvoltage is required for optimal SPTR. We chose $V_{br}$ + 3 V as the operating voltage to balance sensor gain and noise while achieving sub-300 ps FWHM SPTR.

The profile of single photon pulses from the SiPMs exhibits a long tail when no signal processing is applied. Unaddressed, these long tails would cause optical photon pileup or overlap in the signal and obfuscate photon counting. One of the advantages of employing a waveform digitizer for a prototype neutron scatter camera is that raw waveforms can be directly digitized and processed with digital signal processing (DSP). We applied a digital bandpass on the digitized waveforms from the SPTR measurements with the small-scale prototype readout (7 V RF amplifier voltage) and DRS4 digitizer to investigate the effect when DSP was used to remove the slow component of waveforms and shorten the duration of optical photon pulses. For this, the low frequency cutoff was parametrically raised from 200 MHz to 1100 MHz, and a bandpass as narrow as 1100–1200 MHz yielded SPTR less than 300 ps FWHM. This digital bandpass window was applied in the small-scale prototype detector measurements.

### 4.3. Small Scale Prototype Measurements

Variation in the noise response was observed across the SiPM arrays (Figure 8a), which is attributed to variations in breakdown voltage in SiPM elements and need for improved thermal regulation in the experimental setup. Breakdown voltage variation was not controlled for in the construction of the small scale prototype. Future implementations will control overvoltage for variations in breakdown voltage. Room temperature dry air was blown over the front-end electronics, but heat could be transferred to the SiPMs through the inner layers of the prototype board, as it was observed that the SiPMs with increased noise rate were in the row closer to the electronic readout. Nonetheless, the achieved total noise performance does not prohibit the development of a neutron double scatter camera. We also note that there are additional strategies which can be explored to mitigate the total noise photon population in a prototype camera design, such as the implementation of optical bandpass windows that are transmissive to scintillation light, while being absorptive to the longer wavelength emissions of crosstalk photons generated during the avalanche [22]. Other works have shown substantial reductions in detected correlated noise photons in SiPM arrays [23]. We will explore implementation of these optical windows in the development of the prototype camera.

There are multiple methods to identify the true first scintillation photons [24,25] to open a photon counting window. Researchers developing methods for photon counting with digital avalanche photodiodes and digital SiPM arrays have developed methods for determining the true first luminescence photons by exploiting the increase in optical photon density from luminescence relative to the sparse noise counts. Alternatively, the common signal from the summed output can be used to define a region of interest in which optical photon events should be analyzed in each channel. For these analyses, a photon counting window could be defined from the full width of the waveform observed from the common trigger channel.

Note there are several considerations for this proof-of-concept work in regards to the achieved energy and position response of the small scale prototype. Primarily, the aspect ratio of the plastic scintillator, in combination with the absorptive surface finish. These factors result in a significant amount of the luminescence yield being discarded, and edge events will exhibit more light loss than interactions in the center of the detector. Improved aspect ratio in a cubic detection volume would mitigate these effects, especially for full six-sided coverage with SiPM arrays. In addition, deriving a linear position response in monolithic scintillation detectors with analytical methods requires more careful optimization of aspect ratio, surface finish, and reflector properties [26], which is beyond the scope of this work. For these reasons, it is not instructive to extract metrics such as achievable position resolution from the small scale prototype, as they will not translate a fully instrumented prototype camera. The primary goal and outcome of the present work is to identify an SiPM and electronics chain that could facilitate optical photon counting and demonstrate that their integration into a prototype detector enables identification of 3D position and time of interaction, along with energy deposited, on an event-by-event basis.

## 5. Conclusions

We have evaluated Hamamatsu S13360-6075 SiPMs for integration into a monolithic neutron double scatter imager having multiple sides instrumented with detector readout. Expected uncorrelated and correlated noise performance was quantified, and achievable SPTR were measured as a function of applied overvoltage. SPTR was also quantified with different digital bandpass filters that enable time-resolved scintillation photon counting of multiple optical photons detected in SiPM arrays. A small scale prototype detector comprising two sixteen channel SiPM arrays coupled to opposing, narrow ends of a 1.5 × 5.5 × 5 cm${}^{3}$ EJ-240 scintillator was constructed, and achievable noise, energy, and position response were observed. Altogether, the measurements performed with the small-scale prototype demonstrate: Front-end signal processing of a monolithic detector can be instrumented to count individual photons detected in arrays of SiPMs; an acceptable noise level can be achieved for up to six sided readout of a monolithic scintillation volume, corresponding to <100 keV scintillation yield; energy imparted and 3D position of interaction can be derived from scintillation photon counting to explore and develop standoff fast neutron imaging. These findings will guide the development of a prototype neutron double scatter imager constructed from a single monolithic scintillation volume.

## Data Availability

Data can be provided upon request from the corresponding author.
